# Knowledge attitudes and practices of grade three primary schoolchildren in relation to schistosomiasis, soil transmitted helminthiasis and malaria in Zimbabwe

**DOI:** 10.1186/1471-2334-11-169

**Published:** 2011-06-13

**Authors:** Nicholas Midzi, Sekesai Mtapuri-Zinyowera, Munyaradzi P Mapingure, Noah H Paul, Davison Sangweme, Gibson Hlerema, Masceline J Mutsaka, Farisai Tongogara, Godfrey Makware, Vivian Chadukura, Kimberly C Brouwer, Francisca Mutapi, Nirbhay Kumar, Takafira Mduluza

**Affiliations:** 1 National Institute of Health Research, Box CY 573, Causeway Harare, Zimbabwe; 2 College of Health Sciences, Department of Medical Microbiology, P.0 Box A178, Avondale, Harare, Zimbabwe; 3 University of Zimbabwe, Department of Biochemistry, P.O Box MP167, Mount Pleasant, Harare, Zimbabwe; 4 Johns Hopkins Bloomberg School of Public Health, Department of Molecular Microbiology and Immunology, Baltimore, Maryland, USA; 5 Zimbabwe National Statistics Agency PO Box CY 342, Causeway, Harare, Zimbabwe; 6 University of California, San Diego, Division of Global Public Health, Department of Medicine, San Diego, California USA; 7 University of Edinburgh, Institute for Immunology and Infection Research, Edinburgh, UK

**Keywords:** Knowledge, attitudes, practices, schistosomiasis, soil transmitted helminthiasis, malaria

## Abstract

**Background:**

Helminth infection rates in grade three children are used as proxy indicators of community infection status and to guide treatment strategies in endemic areas. However knowledge, attitudes and practices (KAP) of this target age group (8-10 years) in relation to schistosomiasis, soil transmitted helminthiasis (STHs) and malaria is not known at a time when integrated plasmodium - helminth control strategies are being advocated. This study sought to assess KAP of grade 3 children in relation to schistosomiasis, STHs and malaria in order to establish an effective school based health education for disease transmission control.

**Methods:**

Grade 3 children (n = 172) attending four randomly selected primary schools (one in rural and 3 in the commercial farming areas) in Zimbabwe were interviewed using a pre-tested interviewer administered questionnaire. The urine filtration technique was used to determine *S. haematobium *infection status. Infection with *S. mansoni *and STHs was determined using a combination of results from the Kato Katz and formol ether concentration techniques. *P. falciparum *was diagnosed by examination of Giemsa stained thick blood smears.

**Results:**

It was observed that 32.0%, 19.2% and 4.1% of the respondents had correct knowledge about the causes of schistosomiasis, malaria and STHs, respectively, whilst 22.1%, 19.2% and 5.8% knew correct measures to control schistosomiasis, malaria and STHs. Sixty-two percent and 44.8% did not use soap to wash hands after toilet and before eating food respectively, whilst 33.1% never wore shoes. There were no functional water points and soap for hand washing after toilet at all schools. There was a high prevalence distribution of all parasites investigated in this study at Msapa primary school - *S. haematobium *(77.8%), *S. mansoni *(33.3%) hookworms (29.6%) and *P. falciparum *(48.1%). Reports that participant had suffered from schistosomiasis and malaria before were significant predictors of these diseases (p = 0.001 and p = 0.042, respectively). Report that participant had blood in urine on the day of examination was a significant predictor of schistosomiasis (p = 0.045).

**Conclusion:**

There is a critical need for targeting health messages through schools in order to reach the most susceptible schoolchildren. This will empower the schoolchildren with the basic knowledge and skills ultimately protecting them from acquiring schistosomiasis, STHs and malaria.

## Background

More than 2 billion people are affected with schistosomiasis and soil transmitted helminthiasis (STHs) of whom more than 300 million suffer from associated morbidity and 155 000 deaths are reported annually [1]. Most of the morbidity due to schistosomiasis, and STHs is borne by school-age children whose physical, nutritional and cogitative potential are resultantly impaired [1-3]. Approximately 300-500 million malaria clinical cases and one million deaths due to malaria occur annually. Over 90% of the disease burden occurs in Africa south of the Sahara alone [4]. In areas of unstable transmission, malaria accounts for 10 to 20 percent of all-causes of mortality among school-age children [5], and poorer cognitive abilities characterise those who have suffered repeated malaria attacks. A Kenyan study has shown that primary school children miss 11% of school days because of malaria [6].

Schistosomiasis is among the top 14 out patient treated diseases in Zimbabwe [7]. *S. haematobium *is predominantly distributed in all provinces with some areas in the Northern -Eastern region registering up to 80% of the disease [8,9]. The age group 8-15 years recorded the highest national incidence rate of schistosomiasis (8.8 per 1000) in 2002 [7]. Chandiwana and Woolhouse (1991) observed that water contacts were related to age with the highest in the 8-10 year olds [10]. The prevalence of infection was also highest in this age group (67%) compared to others. Soil transmitted helminths have been described in the Eastern highlands and Kariba basin [11-15]. Malaria is a serious public health problem in the country. The disease is distributed in all provinces with malaria top 20 districts registering incidence rates ranging from 114- 401/1000 [7]. *P. falciparum *which is responsible for the most severe and fatal form of the diseases accounts for 97% of malaria infection in the country [16]. Mixed infection with schistosomiasis, STHs and *P. falciparum *in primary school children has been described by Midzi et al, (2008) [17].

The World Health Organisation recommends that before any mass control programme is undertaken, the extent of helminthiasis distribution (prevalence and intensity) must be determined in order to inform programme managers on the best intervention strategies [3,18-20]. It also states that for any baseline survey or community diagnosis, a group of primary school students in Grade 3 (8-10 years) should normally be the study population for the baseline survey and for periodic monitoring and evaluations of intervention strategies because of the epidemiological importance of this age group with respect to STHs and schistosome infections [10,18].

Whilst immense efforts are being made to control morbidity due to helminthiasis and the big three diseases with special emphasis on regular combined school based treatment of primary school children [3], it should be borne in mind that community perceptions, knowledge and practices have a major role in sustainable control interventions. Surprisingly little is known about knowledge, attitudes and practices of grade three children, the most susceptible age group, in relation to causes and control measures for schistosomiasis, STHs and malaria. WHO recommends that any control measure for schistosomiasis and STHs should involve three main components: drug treatment, improved sanitation and health education aimed at reducing transmission and re-infection by encouraging self protective health behaviours [2]. Control of malaria is dependent on case management, vector control by indoor residual spraying to kill mosquitoes and avoidance of contact with vectors by use of mosquito nets and mosquito repellents by people. These measures require individual and community participation through modification of their risk behaviours toward the practices that protect them from infection or re-infection applying basic knowledge about causes and prevention of parasitic diseases.

Zimbabwe has drafted a national plan of action for the control of schistosomiasis and STHs. Ahead of this national programme, a pilot study to determine the effectiveness of combined regular school based de-worming for schistosomiasis, STHs, prompt malaria treatment and health education was conducted in areas endemic for schistosomiasis only and those co-endemic for schistosomiasis, STHs and malaria [17]. During the implementation of the programme, a KAP survey was organized at baseline with the objectives (i) to evaluate grade three children's knowledge about schistosomiasis, STHs and malaria causes as well as their prevention measures (ii) to evaluate personal hygiene practices of this target age group in relation to schistosomiasis, STHs and malaria transmission (iii) to assess the extend to which safe water and sanitary facilities are provided/utilized in the communities and schools and (iv) to determine the association between pupil's experience of the diseases, practices and knowledge of the diseases with parasitology results. The information was used in the pilot programme to plan relevant school based health education activities for the integrated helminthiasis - malaria control programme in selected primary schools and to provide baseline information for the assessment of any changes in KAP following intervention.

## Methods

### Study areas and population

The survey was conducted in grade 3 children (n = 223) living in Nyamaropa rural area (47.1%) that is located in Shamva district, Mashonaland Central Province and Burma Valley commercial farming area (52.9%), which is located in Mutare district, Manicaland Province. The age of the study population ranged from 5 to 15 years and 47.1% were males. The study areas are described in detail elsewhere [17]. Ethical approval was granted by the Medical Research Council of Zimbabwe (Ethical approval reference number, MRCZ/A/993a). Informed parental consent was sought and participation of children in the study was voluntary.

### Study design

The study was a descriptive cross-sectional survey part of a longitudinal intervention study investigating the distribution of polyparasitism with schistosomiasis, STHs and *P. falciparum *among primary schoolchildren in rural and farming areas in Zimbabwe: Impact on anaemia and the effectiveness of regular school based deworming and prompt malaria treatment.

### Sampling scheme

Manicaland Province was conveniently chosen for the study due to its geographical location (Eastern Highlands) characterised by high annual rainfalls, wet soils and malaria endemicity [7]. Multistage sampling technique was used to select the district, ward and schools in which the study was conducted. Using rotary method, Mutare District was randomly selected from 7 districts in the Province, ward 7 was selected from 36 wards in Mutare District and three Primary schools among 5 Primary schools in ward 7 were also selected using the same method. Mashonaland province was also conveniently selected to complement the study participants from a typical rural area. Shamva District and Ward 10 were randomly selected and the only primary school in this ward (Nyamaropa) was included in the study.

### Sample size determination

This is part of a bigger study investigating the impact of school based integrated control of helminthiasis and malaria on anaemia in primary school children. However only grade 3 children (n = 223) were included in the KAP component of the main intervention study.

### Inclusion and exclusion criteria

Only grade 3 children were included in the KAP study. Their KAP were perceived as proxy of their parents/guardians since at this level the children's primary school syllabus does not include such diseases. Children who did not respond to the questionnaire were excluded.

### Knowledge and Practice studies

A questionnaire that contained questions on demographic data, sources of water, sanitary facilities, knowledge of participants about causes and preventive measures for schistosomiasis, STHs and malaria, and practice of children was designed and administered at the beginning of the main study (June 2004). Thus the main outcomes of the study were, knowledge of participants regarding causes and preventive measures of the diseases under investigation, experience (if the participants had suffered from the diseases before) and their practices in relation to schistosomiasis, STHs and malaria transmission.

### Causes, risk factors and preventive measures considered correct for schistosomiasis

The following were considered correct causes and risk factors of schistosomiasis: swimming or bathing in ponds, streams, rivers or dams, playing in the river, contact with snail infested/contaminated water, crossing the river bare foot, washing clothes or dishes in the river, stream or pond, urinating or defecating near or in the river, fishing whilst standing in the river, worms in the river, fresh water snails. The following were considered correct preventive measures for schistosomiasis: avoid swimming, playing or bathing in the pond, stream, river or dam, avoid washing dishes or clothes in the pond, steam, river or dam, kill fresh water snails using chemicals, visit the health care centre for treatment, avoid playing in the river, avoid contact with contaminated water, avoid crossing the river bare foot, cross the river on a bridge, avoid fishing whilst standing in the river and use toilet when defecating or urinating.

### Causes and preventive measures considered correct for malaria

Mosquito and mosquito bite were considered as correct causes of malaria. The following responses in relationship to malaria prevention were considered correct: sleep under ITN, apply mosquito repellents, wear long robes, take prophylactic medicines when visiting malarious area, seek prompt treatment when infected, spray the walls of the house with chemicals to kill resting mosquitoes, fill all holes, ground openings and destroy empty tins that may act as breeding places for vector mosquitoes, burning local herbal plant leaves and clear grass around homes.

### Causes, risk factor and preventive measures considered correct for STHs

The following responses were considered correct causes and risk factors of STHs: Eating with dirty hands, handling fruits with dirty hands, not wearing shoes, leaking dirty fingers, keeping fingernails dirty and long, not washing hands after toilet, not washing hands before eating food, drinking dirty water eating unwashed fruits and vegetables, defecating, using the bush as toilet for defecating. Preventive measures for STHs considered correct included the following: washing hands with soap before eating food, washing fruits in clean water before eating, washing hands with soap after toilet, using toilets when defecating or urinating instead of bush toilet, keeping fingers short and clean, drinking clean water, keeping drinking water protected from flies.

### Parasitological investigations

As part of the main study, participants were screened for *S. haematobium*, *S. mansoni*, soil transmitted helminths and *P. falciparum *using quantitative and qualitative parasitological techniques [17]. The urine filtration technique was used to determine *S. haematobium *infection status. Infection with *S. mansoni *and STHs was determined using a combination of results from the Kato Katz and formol ether concentration techniques. A person was considered positive for *S. mansoni *or STHs (hookworm, *A. lumbricoides *and *T. trichiura*) if *S. mansoni *ova or ova of any of the STHs species were observed using either the Kato Katz or the formol ether concentration technique or if ova were observed in both the Kato Katz slide preparation or the formol ether concentration slide preparation. An individual was considered negative for *S. mansoni*, all or any of the STHs species if no ova were observed in slide preparations made using both techniques. *P. falciparum *was diagnosed by examination of Giemsa stained thick blood smears.

### Data analysis

Data collected were entered and analysed using SPSS 8.0 software package. Chi-square, Fisher's Exact test and Multiple regression analysis were used where appropriate.

## Results

Out of 223 class three participants included in the study 172 (77.1%) responded to the KAP questionnaire. Of the 172 respondents, there were more females, 91 (52.9%) than males 81 (47.1%), χ^2 ^= 7.513, p = 0.006. Demographic data revealed that the ages for the participants ranged from 7-15 years with the mean (SD) and median of 9.8 (1.28) and 10.0, respectively. Of the 172 participants 77.3% were aged 8-10 years. The distribution of grade 3 children by school was 106, 59, 30 and 28 for Nyamaropa (rural area), Valhalla, Msapa and Kaswa primary schools respectively. The main tribe was the Shona (81.3%) followed by the Zezuru (9.3%), Ndau (1.7%), Ndebele (1.2%). Of the 172 participants 88.4% were Christians.

### Schools' amenities

The provisions of the school regarding water supply, sanitation and amenities for hygienic practices are described in Table 1. In all schools there was no water point with running water and soap for hand washing after toilet or before eating food. Schools in the commercial farming areas were situated near the clinic compared to a school in the rural area that was located about 10 km away from Madziwa rural hospital.

**Table 1:** Description of water supply, sanitation, environment and health facilities in schools selected for the study

Condition	Schools in Rural area	Schools in the Commercial farming area		
	Nyamaropa	Valhalla	Msapa	Kaswa
Water source at school	Means of waste disposal	Tape	Borehole	Absent
Type of sanitary facilities	VIPs	Pit latrine	Poor pit latrine	Pit latrine
Wash up point after toilet	Concrete tank present fixed with a tape but with no water	Absent	Absent	Absent
Hand washing soap on basin	Absent	Absent	Absent	Absent
Means of waste disposal	Rubbish dump	Rubbish dump	Absent	Rubbish dump
Nearest health facility	Hospital 10 km	Clinic 3.5 km	Clinic 500 m	Clinic 5 km

### Prevalence of parasites

The prevalence of *S. haematobium*, *S. mansoni*, STHs and *P. falciparum *in the study population stratified by school are shown in Table 2. *S. haematobium *was the most prevalent parasite. An interesting observation was the distribution nature of schistosomiasis, STHs and *P. falciparum *in the same area represented by Msapa primary school, where the prevalence of each parasite investigated in this study was highest compared to other schools. When the study population was stratified by site, *S. haematobium *was higher in the rural area compared to the farming area although there was no statistical significant difference (63.5% vs 57.6% respectively), χ^2 ^= 0.616, p = 0.433., It was also observed that hookworms and malaria were common in the farming area only and the prevalence of *S. mansoni *was higher in the rural area (21.0%) compared to the farming area (14.5%) although there was no significant difference (p = 0.273).

**Table 2:** Prevalence of helminths and *Plasmodium *among grade three primary schoolchildren stratified by school in rural and commercial farming areas, Zimbabwe (2004)

Parasite	Overall		Valhalla		Msapa		Kaswa		Nyamaropa		p. value
	(n)	%	(n)	%	(n)	%	(n)	%	(n)	%	
*S. haematobium*	170	60.6	37	54.1	27	77.8	20	40.0	86	62.8	0.052
*S. mansoni*	164	17.7	37	0.0	27	33.3	18	16.7	82	20.7	0.005
Hookworm	165	7.3	37	5.4	27	29.6	19	10.5	82	0.0	< 0.001
*P. falciparum*	118	21.2	34	20.6	27	48.1	15	33.3	42	0.0	<0.001
*A. lumbricoides*	165	0.0	37	0.0	27	0.0	19	0.0	82	0.0	N/A
*T. trichiura*	165	0.0	37	0.0	27	0.0	19	0.0	82	0.0	N/A

Further analysis was done to determine the difference in prevalence of parasites with sex and age. However there was no significant difference in parasitic infection between males and females. Neither was there any significant difference in parasitic infection between different age groups. Although diagnosed as well, *A. lumbricoides *and *T. trichiura *were not observed in grade three children even in the farming area.

### Sanitary facilities used by participants at home

Responses on where children defecated when at home revealed that of 172 respondents, 12% used bush toilets whilst 88% used toilets: pit latrine, improved ventilated pit latrine (VIP) or water closet. Of the 12% that used the bush toilets, 5.7% were living in the commercial farming area and 20.0% lived in the rural area. Communal pit and VIP toilets were commonly used in the commercial farming areas whilst more of VIP toilets were used in the rural area. Only 4 (4.6%) respondents in the commercial farming area reported that they used water closet type of toilet. The same was not used in the rural area. One school had a dysfunctional water tank for hand washing after using the toilet. Other schools did not have hand washing water points. All schools did not have running water points, soap basins and soap for convenient hand washing after toilet.

### Sources of water for drinking and washing/bathing

After stratifying sources of water by school, it became evident that piped water was predominantly used in the commercial farming areas for drinking/cooking with the highest percentage of use of piped water being recorded for children living in farming areas surrounding Valhalla Primary school (73.0%). The percentage of piped water use by children attending Msapa, and Kaswa primary schools were 53.9% and 40.9% respectively. Children attending Nyamaropa Primary school frequently used boreholes and wells (51.2% and 32.6% respectively). Overall 98.9% of water sources used for drinking and cooking were safe for children living in the farming areas and 84.7% safe drinking water sources were used in the rural area. The percentages of respondents who indicated that they bathed or washed in the river/stream/dam were 74.1%, 72.7%, 45.9, and 31.8% for Msapa, Kaswa, Valhalla and Nyamaropa respectively. Overall, 63.2% of respondents in the farming areas and 36.5% of respondents in the rural area used unsafe water (river, stream/dam) for washing/bathing.

### Knowledge of participants about schistosomiasis, STHs and malaria

Of the 172 grade 3 primary schoolchildren who responded to questions regarding their knowledge about schistosomiasis, STHs and malaria, 87(50.6%) were from the farming area whilst 85 (49.4%) were from the rural area. Of these 109 (63.4%) indicated that they new schistosomiasis. However a larger proportion of children in the farming area said they knew schistosomiasis than those in the rural area (75.9% vs 50.6, χ^2 ^= 11.832, p = 0.001). Ninety-seven (65.4%) participants said they new about malaria and this comprised of a higher proportion of children living in the farming area compared to those in the rural area (85.1% vs 27.1%, χ^2 ^= 58.812, p < 0.001). Overall, 45 (26.2%) children said they knew about STHs and more of these lived in the farming area compared to the rural area.

### Experience of participants regarding schistosomiasis, STHs and malaria

**Figure 1:**
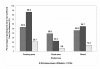
**Proportion of participants who had suffered from schistosomiasis, malaria and STHs**.

Figure 1, shows the proportions of respondents who had suffered from malaria, schistosomiasis and STHs before. Of the 172 respondents, 79 (45.9%) said they had suffered from schistosomiasis. Although there was no significant difference, a higher proportion of children in the farming area indicated that they had suffered from schistosomiasis than those in the rural area (χ^2 ^= 3.418, p = 0.065). Ninety-four children (54.7%) said they had suffered from malaria. A higher proportion of children from the farming area had suffered from the disease than those from the rural area, (χ^2 ^= 70.733, p < 0.001). Only 23 (13.4%) of the respondents said they had suffered from STHs and more of these participants were from a farming area (χ^2 ^= 8.138, p = 0.004).

### Participants' knowledge on causes and prevention measures of schistosomiasis, STHs and malaria

Tables 3 and 4 describe the responses given by participants in relation to causes and control measures of schistosomiasis, STHs and malaria, respectively. Whilst multiple responses were accepted, they were classified into correct or incorrect responses in relation to schistosomiasis, malaria and STHs causes and preventive measures. Of 172 respondents 55 (32.0%, 95%CI: 25.1-39.5) knew the causes of schistosomiasis. There was no significant difference between the proportion of children in the farming area and those in the rural areas who knew the causes of the disease (36.8% vs 27.1, χ^2 ^= 1.869, p = 0.0.172). Thirty-three children (19.2%, 95%CI: 13.6-25.9) correctly new the causes of malaria and this comprised of 26.4% (95%CI: 17.6-37.0) and 11.8 (95%CI: 5.8-20.6) of participants living in the farming and rural areas respectively (χ^2 ^= 5.969, p = 0.015). Only 7 participants (4.1%, 95%CI: 1.7- 8.2) of 172 respondents new the causes of STHs. Of the 172 grade three children who responded to questions regarding the best ways to prevent schistosomiasis, malaria and STHs, 38 (22.1%, 95%CI: 16.1-20.0), 33 (19.2%, 95%CI: 13.6-25.9) and 10 (5.8%, 95%CI: 2.8- 10.4) new the best ways to prevent schistosomiasis, malaria and soil transmitted helminthiasis, respectively (Table 4).

**Table 3:** Responses of grade 3 children regarding their knowledge about causes and risk factors of schistosomiasis, STHs and malaria

Schistosomiasis	n	(%)	Malaria	n	(%)	STHs	n	(%)
No idea	87	50.6	No idea	110	64.0	No idea	155	90.1
Swimming in river or dam	30	17.4	Mosquito/mosquito bite	33	19.2	Dirty water	5	2.9
Playing in the river or dam	14	8.1	Eating with dirty hands	4	2.3	Eat with dirt hands	2	1.2
Drinking dirt water	11	6.4	Hot weather	3	1.7	Playing in dirt water	2	1.2
Eating too much salt	7	4.1	Cold	3	1.7	Playing in the river	2	1.2
Worms in water	6	3.5	Playing in the rain	2	1.2	Drugs	1	0.6
Entering toilet without shoes	3	1.7	Head ache	2	1.2	Infected water	1	0.6
Contact with water	2	1.2	Dirty water	2	1.2	Not eating too much	1	0.6
Snails	2	1.2	Drinking dirty water	2	1.2	Stepping in water	1	0.6
Stepping on urine	2	1.2	Playing in dirty water	2	1.2	Water	1	0.6
Going to the river	1	0.6	Eating maize meal	2	1.2	Eating rotten food	1	0.6
Eating green mangoes	1	0.6	Fishing	1	0.6			
Stepping in vomit	1	0.6	Getting in water	1	0.6			
Jumping over fire	1	0.6	Poor hygiene	1	0.6			
Moving blood	1	0.6	Spontaneous disease	1	0.6			
Not wearing shoes	1	0.6	Swimming in the river	1	0.6			
Stepping in witches place	1	0.6	Toilet	1	0.6			
Urinating in water	1	0.6	Eating green mangoes	1	0.6			
**Total**	**172**	**100**	**Total**	**172**	**100**	**Total**	**172**	**100**

**Table 4:** Responses of grade 3 children regarding their knowledge about causes and risk factors of schistosomiasis, STHs and malaria

Schistosomiasis	n	(%)	Malaria	n	(%)	STHs	n	(%)
No idea	126	73.3	No idea	129	75.0	No idea	156	90.7
Take drugs	18	10.5	Take antimalarial medicines	23	13.4	Take medicines	7	4.1
Avoid playing in water	7	4.1	Visit health care centre	7	4.1	Visit health centre	3	1.7
Avoid swimming in rivers	5	2.9	Wash hands	2	1.2	Don't use dirt water	2	1.2
Visit health centre	5	2.9	Use clean toile	2	1.2	Don't play in water	2	1.2
Avoid too much salt	3	1.7	Drink clean water	1	0.6	Use holy water	1	0.6
Enter toilet wearing shoes	2	1.2	Use mosquito repellents	1	0.6	Don't drink water	1	0.6
Take drugs and herbs	2	1.2	Stay indoors during rain	1	0.6			
Wear shoes	1	0.6	Practice good hygiene	1	0.6			
Drink clean water	1	0.6	Do not play in dirty water	1	0.6			
Boil herbal plant roots	1	0.6	Do not play in rubbish dumb	1	0.6			
Avoid contact with water	1	0.6	Take prophylactic drugs	1	0.6			
			Burn herbal plant leaves	1	0.6			
**Total**	**172**	**100**	**Total**	**172**	**100**	**Total**	**172**	**100**

### Practices of participants in relation to schistosomiasis, malaria and STHs transmission

Of 172 respondents, 98.4% indicated that they ate food with their hands. However 42 (24.4%) said they washed their hands some times before eating food. Only 20 (11.6%) of 172 respondents said they always washed their hands with soap before eating food. Regarding washing of hands after using toilet, 29 (16.9%) said they never washed their hands after using toilet whilst 69 (40.1%) said they sometimes washed their hands after using toilet. Of 143 participants who said they washed hands always or sometimes after using toilet, 89 (62.2%) said they never used soap when washing hands after toilet. Responses of participants regarding their swimming habits showed that 92 (53.5%) swam in the river and of these, 82.6% swam at least once per week. Response regarding use of ITNs revealed that only 15.1% used ITNs. One hundred and fifteen (66.9%) children said they never discussed health issues at home. Of the 57 respondents who said they discussed health issues at home 59.6% said they discussed with their mothers.

### Association of participants' knowledge, experience and practices with urinary schistosomiasis, STHs and malaria Infection

There was a significant association between people who reported that they had suffered from schistosomiasis with *S. haematobium *infection as diagnosed parasitology (OR = 2.60, 95% CI = 1.36-4.96, p = 0.003). Although many respondents who said their urine was red were *S. haematobium *positive, there was no significant association between this response and *S. haematobium *infection based on microscopic examination of urine for schistosome ova. Neither was there any association between swimming in the river with schistosomiasis infection. Malaria infection as diagnosed by microscopy of the thick blood smear was associated with participants' knowledge and experience with the disease (OR = 9.07; 95%CI = 2.02-40.71; p = 0.001 and OR = 19.77; 95%CI = 2.57-152.25; p = < 0.001 respectively). There was no association between, observer's results on spot observation of whether children wore shoes or not, experience of participants with STHs, responses of participants on whether they possessed shoes or not with hookworm infection status.

### Predictors of schistosomiasis and malaria

Multiple logistic regression analysis showed that the reports that participants had suffered from schistosomiasis before and that the participant's urine was red on the day of examination were significant predictors of the disease (p = 0.001 and p = 0.045 respectively).

Although it was of borderline significance, report that participant had urinated near the river was a predictor of schistosomiasis (p = 0.058). Multiple regression analysis also revealed that

having suffered from malaria was a significant predictor of malaria infection (p = 0.042).

## Discussion

There have been a considerable number of studies about the KAP relating to schistosomiasis and malaria in different parts of the world, many of which indicate that misconceptions concerning these diseases still exist and practices for their control have been unsatisfactory [21-26]. Results from our combined schistosomiasis, STHs and malaria KAP study shows that misconceptions about the causes and control of schistosomiasis, malaria and STHs exist in grade 3 children. Some children indicated that eating green mangoes, stepping on witch's place and jumping over fire would cause schistosomiasis. Hot weather, playing in the rain and cold were suggested as causes of malaria. We noted that children in grade 3 have not yet been introduced to lessons on malaria, schistosomiasis and STHs in school (personal communication with grade 3 teachers (2004). Hence knowledge of grade 3 children in Zimbabwe could be a proxy of their parental knowledge or based on whether they have suffered from the disease or not. Misconceptions such as jumping over fire or eating green mangoes cause schistosomiasis (Table 3) could be myths passed on to children by their parents in order to prevent them from real dangers associated with such actions (burning themselves in fire/destruction of fruits before they are ripe).

The fact that a greater proportion of children did not know the causes and prevention measures of schistosomiasis, malaria and STHs (Tables 3 and 4) indicates lack of health education regarding helminthiasis and malaria in grade 3 children. However this is the high risk age group that is capable of transmitting these diseases in the community because of their high risk behaviour and the high prevalence of helminthiasis and plasmodium in the same age- group [10,18,20,27,28]. Whilst there has been great improvement in the control of malaria in Zimbabwe with time allocated for advertising the treatment options for the disease on radio and TV almost every day, it appears that this information is failing to reach this target age group. Probably poor socio-economic status of the rural population where only a few people own either a radio or television set could be a barrier to the flow of health information to school children. Thus the school becomes the strategic existing channel for communicating health information to this most susceptible age group.

Our study revealed that 32.0%, 19.2% and only 4.1% of the respondents knew about the causes of schistosomiasis, malaria and STHs respectively whilst 22.1%, 19.2% and 5.8% knew measures to control the same diseases respectively. Results obtained in Sierra Leone [29] showed that only 30.0% of women of a childbearing age knew that mosquitoes were involved in malaria transmission, while 16.5% of respondents in a Nigerian study had such knowledge [26]. These studies demonstrate knowledge gaps that have to be filled in control of parasitic diseases with health education being an integral component of disease control strategies that should be prioritized in order to motivate the affected populations so that they can change their behaviour towards that which is protective [30]. Children and the population in general lacking knowledge about the causes and prevention measures of helminthiasis and malaria are less likely to take preventive measures to protect themselves from acquiring or transmitting such diseases. For example Vundule and Mharakurwa (1996) showed that the villagers who had poor knowledge of malaria and its causes reported not taking measures of their own to protect themselves [23]. Lukwa et al, (1999) observed that taking mosquito control measures was related to knowledge of malaria transmission, with 24 (75.0%) of those who did not know taking no measures of their own [31]. In a Thailand study, Tomono et al observed that pupils who acquired hookworm infection 8 months after the first stool examination had lower levels of knowledge of STH compared to those who did not [32].

Although in this study swimming in the river/dam was not associated with schistosomiasis and similarly failure to own and use ITN was not associated with malaria, many children (84.9% of 143 respondents) did not sleep under ITNs whilst 53.5% of the 172 respondents said they swam in the river/dam. These practises pre-dispose the target population to malaria and schistosomiasis respectively. Thus, whilst considerable sums of money may be spend on sustained regular treatment of helminths [19] and malaria, as long as the eligible population in at risk communities are not health educated about the endemic diseases and involved in control, poor compliance that militate against success of applied intervention strategies are inevitable [32]. This study has therefore revealed a critical need for targeting health messages towards school age children in order to empower them with the basic knowledge and resources to recognise and manage their health problems.

Results from this study also show that many children discuss health issues with their mothers compared to other community members. This is import since some studies have revealed that most children's main contact with medical care is through their mothers [22]. On the other hand children can act as behaviour change agents in the community since women who are most likely to receive health education from school through their children are more likely to be the motivators and participants in household and community based preventive actions. Results on school amenities (Table 1) revealed another huge gap in relation to school based hygienic practices. One school had a non-functional hand washing water tank whilst the other three schools had no water points for hand washing after using toilet. Neither was there any provision of soap and soap basins at strategic points for hand washing. These observations make us wonder whether it was not a desirability bias of children who claimed that they washed their hands always after toilet and those who claimed that they washed their hands with soap always after using the toilet. Thus provision of water and hand washing facilities in schools to enable hand washing after using toilets and before eating food is the key area to be addressed in order to reduce the transmission of water and sanitation related diseases and helminth infestations in school children. This is because schools are often crowded with children drawn from different communities some of which frequently experience diseases outbreaks that are acquired through oral faecal contamination such as cholera, dysentery as well as STHs.

The findings on the sources of water used by participants for washing showed that a greater proportion of children in the farming area 62.7% and 36.5% of respondents in the rural area used unsafe water for washing and bathing. However the prevalence results for schistosomiasis showed a significantly higher proportion of children infected with *S. haematobium *in the rural area than the farming area (64.8% vs 51.3%, p = 0.044). The reason for these findings could be a desirability bias regarding water source used for washing and bathing by respondents in the rural area [21,22]. However other water contact activities such as watering vegetables, fishing and swimming could have contributed as important risk factors for transmission of schistosomiasis in this age group [10]. Thus heterogeneities in individual water contact behaviour and focality of snail intermediate host distribution could be responsible for the observed differences in prevalence of schistosomiasis in the study population by site and school [10,33].

The demographic data obtained in this study shows that 77.3% of grade 3 children are aged 8-10 years. Chandiwana and Whoolhouse (1991) observed that the prevalence and mean intensity of *S. haematobium *were highest in the 8-10 year age group (67.6%) and 13.1 eggs/10 ml respectively [10]. He also showed in the same study that the 8-10 year age group recorded the highest water contact activities than other age groups. Kloos et al, (1983) observed that young children 6-8 year old had higher exposure index in water, 61.3 vs 30.0 for the 12-14 years old [34]. Thus, grade 3 children are a high risk age group for schistosomiasis in endemic communities [3,18].

Table 2 shows significant differences in the distribution of parasites among schools representing different community clusters. It also shows the clustering of schistosomiasis, STHs and *P. falciparum *in the same school (Msapa). These results could be contributed by the high risk behaviour of the community as a higher proportion of children from Msapa Primary school reported that they bathed or swam in the river. The clustering effect of helminths and *Plasmodium *in the same, school, house holds or individuals has significant implications on co-morbidity and severity of diseases [35-37]. On the other hand the clustering of diseases in the same community and households provides a conducive platform for the cost effective integrated control of neglected tropical diseases with the big three (malaria, TB and HIV/AIDS) and also justifies preventive chemotherapy currently being advocated by the World Health Organisation [19,28,38,39]. Cost effective combined health education about these diseases can also be delivered to the target age group at the same time through the already existing school channels.

## Conclusion

In conclusion, this study has demonstrated a critical need for targeting health messages through schools in order to reach the most susceptible schoolchildren. This will empower high risk age group with the basic knowledge and skills ultimately protecting them from acquiring schistosomiasis, STHs and malaria.

## Competing interests

The authors declare that they have no competing interests.

## Authors' contributions

TM, NM, NK, KCB, FM contributed to the concept and design of the study protocol; NM, SZ, DS, NHP, GH, FT, MJM, VC and TM carried out the clinical assessment and parasitology; TM, NM, MG, and MPM carried out the analysis and interpretation of the data; NM and TM drafted the manuscript. All authors read and approved the final manuscript.

## Pre-publication history

The pre-publication history for this paper can be accessed here:

http://www.biomedcentral.com/1471-2334/11/169/prepub
